# A National Consensus Forum on improving cornea donation and transplantation access in Canada

**DOI:** 10.1186/s12919-021-00215-6

**Published:** 2021-05-11

**Authors:** Steven S. Bae, Guillermo Rocha, Christine Humphreys, Clara C. Chan, Sonia N. Yeung

**Affiliations:** 1 Department of Ophthalmology and Visual Sciences, University of British Columbia, Vancouver, British Columbia Canada; 2 Department of Ophthalmology, University of Manitoba, Winnipeg, Manitoba Canada; 3 Eye Bank of Canada - Ontario Division, Toronto, Ontario Canada; 4 Department of Ophthalmology and Vision Sciences, University of Toronto, Toronto, Ontario Canada; 5 Eye Bank of British Columbia, Vancouver, British Columbia Canada

**Keywords:** Organ donation, Corneal transplantation, Eye bank, Meeting report, Access

## Abstract

A consensus meeting was held in Toronto on February 9–10, 2020 to discuss ways to improve cornea donation and transplantation access in Canada. The meeting brought together eye and tissue bank representatives, health authority and hospital leadership, transplant ophthalmologists, organ donation organizations, transplant recipients, donor families and several national organizations. Through facilitated discussions in multidisciplinary, gender-balanced, and geographically balanced small groups, participants identified opportunities for improvement in the Canadian cornea donation and transplantation system. Discussion occurred around broad themes of donor tissue demand, supply, access, utilization, interprovincial sharing and cost recovery, interprovincial knowledge sharing and research. This event marked the first time in 10 years in which the Canadian cornea transplantation community came together.

## Introduction

The demand for corneal transplantation is increasing, but the supply of donor corneas has not changed in Canada over the last 6 years [1]. There is little quantitative data regarding the number of patients waiting for cornea transplants or their actual wait times. Some provinces have excess cornea supply, but there is no funding to support costs associated with tissue recovery, processing, and distribution between provinces. As a result, interprovincial sharing of donor corneas is infrequent. Even within existing eye bank infrastructure, the cornea transplant community sees a lack of funding as a barrier to the number of donor corneas processed. Availability of operating room time is another barrier to accessing transplantation surgery. Although advances in eye banking have enabled the use of eye bank prepared donor tissue, many ophthalmologists spend valuable operating room time processing corneal tissue. These and many more topics relevant to corneal donation and transplantation access were the subject of much needed discussion and collaboration on a national level. The 2020 National Consensus Forum on Improving Cornea Donation and Transplantation Access in Canada was held in Toronto from February 9–10, 2020 to bring together an interdisciplinary community to advance the goal of improving access to corneal transplantation nationally.

## Methods

A total of 44 participants including eye and tissue bank representatives, health authority and hospital leadership, transplant ophthalmologists, organ donation organizations, transplant recipients, donor families and several national organizations – including the Canadian Ophthalmological Society, the Canadian National Institute for the Blind, the Canadian Donation and Transplantation Research Program, the Canadian Standards Association – Ocular Technical Committee, Canadian Blood Services, and the Donation Physician Network — participated in the forum. International perspectives were also sought, with representation from Australia. Family members of donors as well as transplant recipients also shared their experiences as important members of the transplant community.

In order to promote informed discussion, participants received information obtained from several surveys completed by Canadian eye banks, organ donation organizations, and corneal transplant surgeons. In addition, participants were provided information on the current provincial legal statutes and frameworks pertaining to corneal donation and transplantation. Literature reviews with data analysis and current practices were summarized for each major discussion topic.

Discussions were facilitated around demand, supply, access, utilization, interprovincial sharing and cost recovery, and interprovincial knowledge sharing and research. Participants were divided into multi-disciplinary, gender and geographically balanced groups in a World Café format. The World Café methodology is a recognized effective and flexible format for hosting group dialogue, which facilitates collaborative conversation, sharing of knowledge, and possibility for action in groups of various sizes [2].

### World Cafés

#### World Café: demand

The World Café on “Demand” was led by Dr. Sonia Yeung (Medical Director, Eye Bank of British Columbia). Due to lack of data on current waiting lists, ophthalmologists and eye banks identified a difficulty in matching supply to demand. With that said, the 2019 survey of Canadian eye banks revealed 1 in 3 transplant surgeons estimated over 1 year waiting time for non-urgent transplants. Potential opportunities for improvement that were discussed included: creating a standardized data system to monitor waiting times for assessment and surgery, highlighting patient stories to demonstrate the impact of long wait times and to generate political interest, and forming an advisory committee with appropriate government representation to maintain accountability and promote ongoing discussions.

#### World Café: supply

The World Café on “Supply” was led by Mike Bentley (Manager, Provincial Initiatives, Alberta Health Services). It was noted that the number of corneal transplants has been stagnant over the last 6 years, with most provinces having to import cornea tissue from the United States. There was strong consensus in desire for Canadian eye banks to become self-sufficient in cornea tissue supply. Identified opportunities for improvement included: education for health professionals in donor identification and referral, and routinely offering patients the ability to donate. Furthermore, there was interest in optimizing an interprovincial donor cornea sharing strategy.

#### World Café: access

The World Café on “Access” was led by Dr. Clara Chan (Medical Director, Eye Bank of Canada Ontario Division). The 2020 Canadian eye bank survey revealed that 40% of transplant surgeons felt referral times needed improvement, and 75% of eye banks felt wait times for corneal transplant needed improvement. There were wide ranges of waiting times for surgery depending on province. Suggestions were raised including a working group to track and monitor provincial demand for donor tissue, and developing national standards, for example, on graft acceptance criteria and patient prioritization strategies. A national waitlist was not supported as urgent patients are typically able to receive timely transplants. It was noted that in addition to graft supply, operating room time and individual surgeon volume allocation were also barriers to increasing transplantation numbers.

#### World Café: utilization

This World Café was led by Christine Humphreys (Director, Eye Bank of Canada Ontario Division). There is known variability amongst transplant surgeons and eye banks on minimum suitability criteria, which affect utilization rates. Recent Canadian utilization rate has been measured at 90% [1]. Opportunities raised included development of a national minimum suitability criteria for donor tissue and transplant corneas, and development of a national data registry on utilization rates with benchmark targets. Furthermore, scheduling transplant surgeries over the week was suggested to reduce challenges of tissue expiry.

#### World Café: interprovincial sharing and cost recovery

This World Café was led by Etienne Fissette (Director, Human Tissue Operations, Héma-Québec). Given that some provinces are self-sufficient in supply and have the ability to increase supply, interprovincial sharing would reduce the need to import corneas from the United States. A centralized system for communicating both the need and availability of tissue was suggested to facilitate interprovincial sharing. Development of cost recovery methods was seen as an important component to allow provinces to donate corneas to other regions. An identified barrier to interprovincial sharing was that not all Canadian eye banks are accredited by the Eye Bank Association of America (EBAA), which restricts distribution of corneas from only accredited eye banks or those eye banks that follow EBAA standards.

#### World Café: interprovincial knowledge sharing and research

This World Café was led by David Hartell (Associate Director, System Development, Canadian Blood Services). A gap in a national approach to professional education for health care workers in ocular tissue donation was identified, and a suggestion was made to create an evidence-based curriculum to address this gap. Furthermore, participants felt a lack of research and innovation in the Canadian eye bank community. By collaborating with the Canadian Ophthalmological Society and the Canadian Donation and Transplantation Research Program, there could be opportunities for funding. Developing an interdisciplinary community of practice was identified as important to share information broadly and to inform national guidance and education programs.

### Consensus building exercise

Following these World Cafés, a consensus building exercise was undertaken to envision the ideals of the Canadian cornea donation and transplantation system. A vision statement, mission statement, and recommendation statement were drafted by the patient and family partners, further discussed and refined with all Forum participants, and finally endorsed collectively (Table 1). Interprovincial collaboration, as well as communicating as a national coordinated voice with governments, stakeholders, and the public at large were identified as key goals for the community.

**Table 1:** Vision statement, mission statement, and recommendation statement from the 2020 National Consensus Forum on Improving Cornea Donation and Transplantation Access in Canada

Vision Statement	Mission Statement	Recommendation Statement
A sustainable patient-centered cornea donation and transplantation system which optimizes, aligns, and coordinates provincial program activities.	To provide Canadians with the opportunity to give the gift of vision at end of life, and to equitably share this gift. To support donors, recipients, and their loved ones. To champion the technicians, surgeons, and support staff who make this gift possible. To fully respect the gift by optimizing the utilization and utility of all donated tissue.	To create a Canadian cornea donation and transplantation system that is self-sufficient and eliminates corneal transplant waiting lists within 5 years.”

A number of actionable recommendations emerged from the consensus building exercise to achieve these goals. In broad categories, they include: creation of an advisory committee, fostering a community of practice, development of a national data strategy, interprovincial cornea sharing, alignment with the broader organ donation and transplantation community, continued engagement of patient and donor families, partnership with government, increasing public awareness and professional education, and development of a national research network. There was significant enthusiasm among participants to move these actionable items forward.

## Conclusions

In summary, the “2020 National Consensus Forum on Improving Cornea Donation and Transplantation Access in Canada” proved to be a meaningful event that engaged a multidisciplinary group and culminated in actionable tasks to improve the status of cornea donation and transplantation in Canada. There was positive feedback from the event, with an average rating of 4.7/5 regarding the success of the forum in meeting its objectives. All patient partner participants indicated a positive experience and feeling like they benefited from their participation in the forum. The full meeting report is available online [3].

## Abbreviation

EBAA: Eye Bank Association of America
